# Correlation between change in serum creatinine concentration and renal cortical anisotropic backscattering artifact in azotemic cats during hospitalization

**DOI:** 10.1080/01652176.2024.2384910

**Published:** 2024-07-31

**Authors:** Ming-Jen Kang, Pin-Chen Liu, Hock Gan Heng, Kuan-Sheng Chen

**Affiliations:** a Department of Veterinary Medicine, College of Veterinary Medicine, National Chung Hsing University, Taichung, Taiwan; b Veterinary Medical Teaching Hospital, College of Veterinary Medicine, National Chung Hsing University, Taichung, Taiwan; c VetCT, Orlando, FL, USA

**Keywords:** Clinical outcome, cortical echogenicity, ultrasound, hospitalization, azotemia

## Abstract

Information on the clinical outcomes of feline azotemia using ultrasound examinations is limited. This study aimed to understand the correlation between cortical anisotropy backscattering artifact (CABA) and serum creatinine (sCr) changes in feline azotemia after hospitalization and to investigate whether CABA is useful for predicting the clinical outcome of feline azotemia. Sixty-five hospitalized cats with azotemia, including 49 cats with moderate or severe azotemia (severe group) and 16 cats with mild azotemia (mild group). This retrospective study reviewed the CABA using ultrasound images of cats hospitalized with azotemia between 2016 and 2021. The correlation between CABA and the clinical outcomes of cats with azotemia was investigated using the chi-squared or Fisher’s exact test, and the intra- and inter-observer agreements in CABA were assessed using McNemar’s and Cohen’s kappa tests. The presence of CABA was significantly positively correlated with the clinical outcomes of cats with azotemia only in the severe group (*p* = 0.0034, odds ratio = 8.57). There was no association between CABA and clinical outcomes in cats with mild azotemia (*p* = 0.75). CABA can be used for clinical outcome prediction in moderate and severe feline azotemia, with a sensitivity of 80.8% and a specificity of 73.9%. Also, satisfactory intra- and inter-observer agreements were revealed in the detection of CABA during ultrasound image review. Our study demonstrated that cats with moderate and severe azotemia with CABA observed during ultrasonography might have better clinical outcomes. These findings provide additional information on the prognosis and treatment of feline azotemia.

## Introduction

Azotemia is characterized by elevated levels of non-protein nitrogenous compounds, such as blood urea nitrogen (BUN) and serum creatinine (sCr) due to decreased glomerular filtration rate (GFR) and difficulty excreting nitrogenous waste (Dibartola and Westropp 2020). GFR is usually indirectly estimated by other renal biomarkers like sCr because direct estimation is not feasible in routine veterinary practice; however, it is the most sensitive indicator of renal function (Kongtasai et al. 2022). In cats, an elevation of sCr over 1.6 mg/dL is considered azotemia, according to the International Renal Interest Society (IRIS). Based on the etiology, azotemia is classified into three categories: pre-renal, intrinsic, and post-renal (Dibartola and Westropp 2020).

Complete abdominal ultrasound examination is recommended for patients with azotemia in small animal practice to aid in diagnosis, treatment planning, and prognosis evaluation. Ultrasonographic findings associated with feline azotemia were described as perinephric fluid, renal volume reduction, renal cortical hyperechogenicity, and loss of corticomedullary differentiation. These findings could also be found in intrinsic renal problem. In post-renal azotemia, findings such as ureteral calculi with obstruction, and dilated renal pelvis were observed (Lamb et al. 2018). Among these abnormal features, increased cortical echogenicity is the most common finding in various renal disorders (Debruyn et al. 2012). Associations have been demonstrated between the hyperechoic cortex and histopathological lesions, including interstitial inflammatory infiltration (interstitial nephritis), tubular necrosis, and fibrosis (Zotti et al. 2015). The tendency of greater mean gray value in the cortex toward more severe renal degeneration is also reported (Banzato et al. 2017). Besides these findings, human studies even illustrated the positive correlation between renal echogenicity and sCr level (Siddappa et al. 2013). However, routine cortical echogenicity assessment in cats is based on subjective comparison with adjacent organs, such as the liver and spleen (Yabuki et al. 2008). False assessments can occur due to renal fat deposition and various organic echogenicity in different individuals (Lamb et al. 2018). Owing to the challenges mentioned above, the application of cortical anisotropy backscattering artifact (CABA), an alternative method for evaluating the cortical echogenicity of chronic kidney disease (CKD) and renal dysfunction in cats, has been reported in a recent study (Chou et al. 2021). It is described as a phenomenon involving the poles of the renal cortex where the ultrasound beams perpendicular to the renal tubules became more hyperechoic than the regions where the ultrasound beams are parallel to the renal tubules (Ruth et al. 2013). The absence of CABA has been found to correlate with the staging of feline CKD (Chou et al. 2021).

To the best of our knowledge, the use of CABA as a predictor of clinical outcomes in cats with renal disorders has not yet been described. This study aimed to evaluate whether CABA can be used to predict clinical outcomes after hospitalization in cats with azotemia. We hypothesized that the presence of CABA in cats with azotemia may result in an effective decrease in sCr after hospitalization.

## Materials and methods

This retrospective study evaluated renal ultrasound images of cats with azotemia. All cats were hospitalized at the Veterinary Medical Teaching Hospital, National Chung Hsing University (VMTH-NCHU), between June 1st, 2016, and December 31st, 2021. Azotemia was defined as cats with sCr equal to or over the International Renal Interest Society (IRIS) threshold of 1.6 mg/dL.

### The criteria of case selection

Cats were included in the study if they met the following criteria: bilateral renal ultrasonography was performed during hospitalization, sCr data at admission and discharge were provided, the duration of hospitalization was at least 48 h, except for those who died within 48 h, and all cats received intravenous fluid therapy (lactated Ringer’s solution) to maintain hydration during hospitalization. The amount of fluid required for rehydration (ml/kg/24h) was calculation using the following formula: body weight (kg) × dehydration (%) × 1000. The exclusion criteria included severe deformation of the renal cortical structure, such as severe hydronephrosis, neoplasia, polycystic kidney disease, and poor-quality images, making it difficult to evaluate the renal cortex.

### Ultrasonographic procedures

All renal ultrasound examinations were performed in dorsal or lateral recumbency using a 7.2–14 MHz linear transducer (PLT-1204 BT, Toshiba Medical Systems Corporation, Otawara-shi, Japan) or an 8–10 MHz micro convex transducer (PLT-1204 BT, Toshiba Medical Systems Corporation, Otawara-shi, Japan) connected to a commercial ultrasonographic scanner (Xario SSA-660A, Toshiba Medical Systems Corporation, Japan). All examinations were performed by several NCHU-VMTH radiology residents or radiologists with 20 years of ultrasonographic experience.

### Renal images analyses

**Figure 1.**
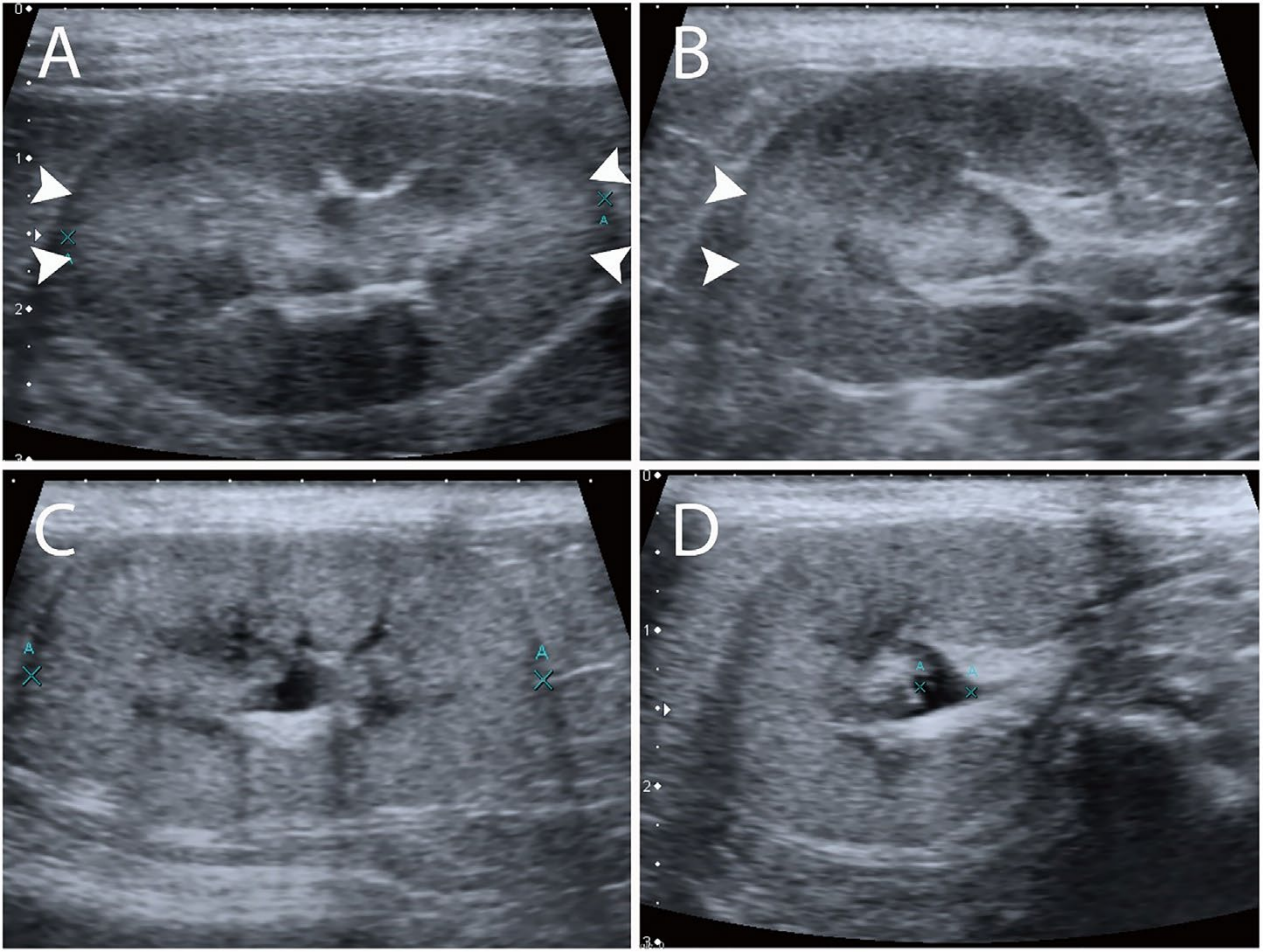
Presence of renal CABA in the longitudinal (A) and transverse (B) planes. Presence of a focally increased echogenicity cortex at the 3 and 9 o’clock of the renal cortex (arrow heads). Absence of renal CABA in the longitudinal (C) and transverse (D) planes.

All ultrasonographic images and cine loop clips of each patient’s kidney with both transverse and longitudinal planes were reviewed by two experienced observers: a 20-year experienced radiologist (Observer 1) and a 2-year experienced radiology graduate student (Observer 2). The observers were blinded to patients’ history, information, biochemical profile, final diagnosis, and ultrasound imaging findings. Both observers reviewed all images with the same digital workstation using Picture Archiving and Communication System (PACS) software (SoliPACS^™^ Web Viewer, EBM Technologies., Taipei, Taiwan) on the same monitor. Renal images were classified as “present” when focal hyperechogenicity was found at the regions close to 3 and 9 o’clock of the renal cortex where the incident ultrasound beams were perpendicular to the renal tubules (in transverse or longitudinal planes) (Figure 1A, B); in contrast, images were classified as “absent” when no focal hyperechogenicity at the regions was found (Figure 1C, D), as described previously (Chou et al. 2021). The CABA status of both the left and right kidneys was recorded. As for the total CABA status of the case, “present” was recorded when only a single side of the renal CABA or both appeared. Both observers interpreted the images again after 2 weeks from the first interpretation to minimize recall bias.

### Grouping and clinical outcome evaluation

The decrease percentage of sCr were evaluated between the renal CABA presence and absence groups during hospitalization. The patients were also categorized based on their sCr decrease percentages into the following groups: those with no decrease or increase in sCr concentration, those with a decrease of >0% but <25%, those with a decrease of >25% but <50%, those with a decrease of >50% but <75%, and those with a decrease of >75%. Moreover, all samples were also divided into two groups based on the serum sCr concentration on the day of admission before treatment. Patients with sCr > 2.8 mg/dL, regarded as IRIS stage 3 with moderate azotemia, or even higher stages with severe azotemia, were assigned to the severe group. The mild group included patients with 1.6 < sCr ≤ 2.8 mg/dL at IRIS stage 2 with mild azotemia. The clinical outcomes of azotemia were evaluated according to the sCr concentration on the day of discharge. Individuals who died during hospitalization were considered to have poor clinical outcomes. In the severe group, a “good” clinical outcome was determined when the sCr level fell below 2.8 mg/dL. As for the mild group, a “good” clinical outcome was determined as the sCr level fell below 1.6 mg/dL. All samples were also separated into CABA “presence” group and CABA “absence” group by the renal ultrasonographic results.

### Statistical analyses

All data analyses were performed using commercial statistical software SAS (version 9.4; SAS Institute, Inc., Cary, North, USA). Two-tailed tests were used in all statistical analyses, and a P-value < 0.05 was considered statistically significant.

The distribution pattern of continuous variables (age, body weight, and sCr) was assessed using the Shapiro-Wilk test. Student’s t-test was used in normally distributed data. In contrast, the Mann-Whitney U test was used in those that did not meet the normal distribution while comparing the continuous variables and decreased sCr percentage between the CABA “presence” and “absence” groups. The Cochran-Armitage trend test was employed to assess whether the increase in CABA presence percentage was in accordance with the classification of sCr decrease percentage. The chi-square or Fisher’s exact test examined the association between categorical variables (sex, reproductive status, and breed) and CABA status. McNemar’s test and Cohen’s kappa were used to verify inter- and intra-observer agreements. The reliability level of ­agreements was classified into five grades based on the Kappa value: < 0 = poor, 0–0.20 = slight, 0.21–0.40 = fair, 0.41–0.60 = moderate, 0.61–0.80 = substantial, and 0.81–1 = almost perfect (Landis and Koch 1977). The chi-squared or Fisher’s exact test was used for the association between the presence of CABA and the clinical outcome of both groups after hospitalization. Univariate and multivariate logistic regression analyses were used to investigate the correlation between the clinical outcome of azotemia in both groups and the independent variables, including breed, age, body weight, sex, reproductive status, and renal CABA status.

## Results

Complete renal ultrasound examinations were performed in 94 hospitalized cats with azotemia during the retrospective study period. Eighteen of the patients were excluded due to incomplete records of signalments; additionally, three were excluded due to short hospitalization time (< 48 h) considered insufficient for efficient treatment. One patient was excluded due to unavailable follow-up data after referral. Six cats were excluded due to uninterpretable renal cortical structure damage (three with polycystic kidney disease and four with renal neoplasia). Finally, 65 cats met the inclusion criteria for the statistical analyses. Of the 65 cats, 56 (86.2%) were discharged, and 9 (13.8%; eight in the severe group and one in the mild group) died during hospitalization. Fifty-six cats (86.2%) were examined using a linear probe, while nine (13.8%) were examined using a micro-convex transducer.

### Signalments

Of the 65 cats, the most common breed was the domestic short hair, accounting for 44 (44/65, 68%) cases. The remaining 21 (21/65, 32%) pedigree cats included four American shorthairs, four Chinchillas, four Persians, and one each from the Bengal, British Curl, Himalayan, Ragdoll, and Siamese breeds. There were 42 males (neutered: 32; 76%) and 23 females (spayed: 21; 91%). The median age of the cats was 9 years (range, 9 months to 20 years). The median body weight of the cats was 3.6 kg, ranging from 1.8 to 7.5 kg.

### Clinical outcomes and the causes of azotemia

The distribution of all the causes of azotemia is shown in Tables 1 and 2. The cats in this study could be affected by more than one cause of azotemia. In this study, feline idiopathic cystitis, urinary tract infection, cystic calculi, and urethral plugs were found in 23 cats, including 22 in the severe group and one in the mild group. In the severe group, sixteen of the 22 (72.7%, 16/22) cats had good clinical outcomes after treating the underlying cause of the post-renal azotemia. The remaining six cats with poor clinical outcome all suffered from intrinsic renal causes simultaneously, even after treating the underlying cause of the post-renal azotemia.

**Table 1.** Causes of azotemia in the severe group.

Causes of azotemia	Renal CABA	Clinical outcome	
		Good	Bad
In (n = 6)	Presence	1	1
	Absence	1	3
Pre (n = 1)	Presence	1	0
	Absence	0	0
Post (n = 6)	Presence	5	0
	Absence	1	0
In and Pre (n = 20)	Presence	5	3
	Absence	2	10
In and Post (n = 2)	Presence	1	0
	Absence	0	1
Pre and Post (n = 7)	Presence	6	0
	Absence	1	0
In, Pre and Post (n = 7)	Presence	2	2
	Absence	0	3

In, intrinsic azotemia. Pre, prerenal azotemia. Post, post-renal azotemia.

**Table 2.** Causes of azotemia in the mild group.

Causes of azotemia	Renal CABA	Clinical outcome	
		Good	Bad
In (*n* = 3)	Presence	0	2
	Absence	0	1
Pre (*n* = 2)	Presence	1	1
	Absence	0	0
In and Pre (*n* = 10)	Presence	0	5
	Absence	0	5
Pre and Post (*n* = 1)	Presence	0	0
	Absence	1	0

In, intrinsic azotemia. Pre, prerenal azotemia. Post, post-renal azotemia.

### Intra- and inter-observer agreements

According to McNemar’s and Cohen’s Kappa tests, the intra-observer reliability evaluations of reviewing the renal CABA images presented significant agreements between the two experienced observers (Observer 1: *p* = 1, Observer 2: *p* = 0.63; McNemar’s test) and showed the Cohen’s Kappa values of 0.83 (Observer 1) and 0.73 (Observer 2), demonstrating an almost perfect (0.81–1) and substantial (0.61–0.80) agreements.

The inter-observer reliability evaluation of renal CABA showed significant agreement in the first (*p* = 0.06, McNemar’s test) and second (*p* = 0.33, McNemar’s test) time interpretation. Cohen’s kappa values both showed substantial (0.61–0.80) agreements between the two observers (first: κ = 0.71, second: κ = 0.74).

The image assessment performed by Observer 1 was chosen for subsequent analysis. In this study, the prevalence of CABA was 55.4% (36/65).

### Differences between the CABA “presence” and “absence” groups

Significant differences have been found between the CABA “presence” and “absence” groups regarding age (Mann-Whitney U test, *p* = 0.01) and body weight (Mann-Whitney U test, *p* = 0.001). Younger age and heavier body weight were observed in cats with CABA. Neither of the categorical variables, including sex (chi-squared test, *p* = 0.36), reproductive status (chi-squared test, *p* = 0.82), or breed (chi-squared test, *p* = 0.74), dehydration status on admission day (chi-squared test, *p* = 0.74), were significantly different between the two groups. Additionally, the length of hospital stay did not differ between the two groups (Mann-Whitney U test, *p* = 0.60) (Table 3).

**Table 3.** Demographic data of the study population in the CABA “presence” and “absence” group.

	Total number of cats (*n* = 65)	CABA “presence” (*n* = 36)	CABA “absence” (*n* = 29)	*p*
*Age (year)*				
median (range)	9 (0.75－20)	6 (0.75－20)	10.93 ± 5.73^#^ (2－20)	0.013*
*BW (kg)*				
median (range)	3.6 (1.8－7.5)	3.85 (2.35－7.5)	3.3 (1.8－7.1)	0.0012*
*Sex*				
Male	42	25	17	
Female	23	11	12	0.36
*RS*				
Intact	12	7	5	
Neutered	53	29	24	0.82
*Breed*				
DSH	44	25	19	
Pedigree	21	11	10	0.74
*DSA*				
Dehydrated	48	26	22	
Euhydrated	17	10	7	0.74
*LOS (day)*				
median	7	7	6.52 ± 2.68^#^	
(range)	(1－35)	(3－35)	(1－14)	0.60

* A statistically significant difference *p* < 0.05 based on the Mann-Whitney *U* test and chi-square test, there was a statistically significant difference between the two CABA status groups. BW, body weight. RS, reproductive status. DSH, domestic short-air. DSA, dehydration status on admission day. LOS, length of hospital stay.

^#^ Mean ± SD was used in the age and length of hospital stay of the CABA “absence” group according to the result of the Shapiro-Wilk test (*p* = 0.073 and 0.50).

After hospitalization, both CABA “presence” (Mann-Whitney U test, *p* < 0.001) and “absence” (Mann-Whitney U test, *p* = 0.002) groups showed a significant decrease in sCr. The significant difference showed that the CABA “absence” group had a significantly higher sCr level on the day of discharge (Mann-Whitney U test, *p* < 0.001) than the CABA “presence” group; however, this result did not apply on the day of admission (Mann-Whitney U test, *p* = 0.21) (Table 4).

**Table 4.** Serum creatinine data of the study population in the CABA “presence” and “absence” group.

	Total number of cats (*n* = 65)	CABA “presence” (*n* = 36)	CABA “absence” (*n* = 29)	*P*
**SCr on the day of admission (mg/dL)**				
median (range)	5.7 (1.7－24.8)	4.8 (1.7－21)	7.1 (2.2－24.8)	0.21
**SCr on the day of discharge (mg/dL)**				
median (range)	2.4 (0.9－12.7)	1.8 (0.9－6.5)	3.7 (1.2－12.7)	<0.0001*
***p***	<0.0001*	<0.0001*	<0.0001*	

* A statistically significant difference *p* < 0.05 Based on the Mann-Whitney *U* test, a statistically significant difference was observed between the two CABA status groups. sCr, serum creatinine.

The percentage decrease in sCr was statistically significant in the group of CABA presence (*p* = 0.029, Mann-Whitney U test) when compared to the group without CABA presence (Table 5). Moreover, there was a significantly higher frequency of renal CABA presence in relation to the classification of decrease percentages between CABA presence and absence groups (*p* = 0.019, Cochran-Armitage trend test, Table 6).

**Table 5.** Serum creatinine decrease percentage in the CABA “presence” and “absence” group.

	Total number of cats (*n* = 65)	CABA “presence” (*n* = 36)	CABA “absence” (*n* = 29)	*p*
**sCr decrease %**				
median (range)	43.9 (93.1－-60.0)	57.7 (92.75－-60.0)	29.9 ± 29.5 (93.1－-36.4)	**0.029** *

* A statistically significant difference *p* < 0.05 based on the Mann-Whitney U test.

**Table 6.** Classification of serum creatinine decrease percentage in the CABA “presence” and “absence” group.

	Presence of CABA (%)	Absence of CABA (%)	P
Crea↓< =0%	4/36 (11.11%)	5/29 (17.24%)	
Crea↓>0%, <25%	6/36 (16.67%)	9/29 (31.03%)	
Crea↓>25%, <50%	7/36 (19.44%)	9/29 (31.03%)	
Crea↓>50%, <75%	8/36 (22.22%)	3/29 (10.34%)	
Crea↓>75%	11/36 (30.56%)	3/29 (10.34%)	
Total number	36	29	**0.019** *

* A statistically significant difference *p* < 0.05 based on Cochran–Armitage trend test.

### Clinical outcome and renal CABA

Sixteen (16/65, 24.6%), 11 (11/65, 16.9%), and 38 (38/65, 58.5%) cats had mild (stage II), moderate (stage III), and severe (stage IV) azotemia, respectively. Forty-nine cats with moderate or severe azotemia were assigned to the severe group, and the remaining 16 cats with mild azotemia were assigned to the mild group.

In the severe group, 53.1% (26/49) of the cats had good clinical outcomes after hospitalization, and seven (7/49, 14.3%) recovered from azotemia. Twenty-one cats (21/26, 80.8%) exhibited CABA. Eight cats died during hospitalization; however, only two out of the eight cats showed the presence of renal CABA. A statistically significant association was found between the presence of renal CABA and the clinical outcome of cats in The severe group (chi-square test, *p* < 0.001; odds ratio = 11.9). When renal CABA was used for clinical outcome evaluation, the sensitivity and specificity were 80.8% and 73.9%, respectively (Table 7).

**Table 7.** Azotemia clinical outcomes evaluation by the presence or absence of renal CABA in different groups.

Groups	Renal CABA	Clinical outcome		*p*	OR
		Good	Bad		
Severe group (*n* = 49)					
	Presence	21/26 **(80.8%)**	6/23 (26.1%)		
	Absence	5/26 (19.2%)	17/23 **(73.9%)**	**0.0001** *	11.9
Mild group (*n* = 16)					
	Presence	1/2 (50%)	8/14 (57.1%)		
	Absence	1/2 (50%)	6/14 (42.9%)	1	0.75

^*^ A statistically significant association (*p* < 0.05, in bold) between renal CABA status and the clinical outcome of azotemia, determined by the chi-square test. Bold percentages (%) in the severe group represent the sensitivity and specificity of renal CABA as a clinical outcome prediction method for azotemia.

In all 16 cats from the mild group, only two (2/16, 12.5%) had reversed azotemia (sCr < 1.6 mg/dL) and a good clinical outcome after hospitalization. One cat died during hospitalization, and renal CABA was present. In contrast to the results in the severe group, no statistically significant association was found between renal CABA and clinical outcomes in the mild group (chi-squared test, *p* = 1) (Table 7).

### Correlations of the independent variables (including breed, age, body weight, sex, reproductive status, CABA status, dehydration status on admission day, and length of hospital stay) and the clinical outcome

Using univariate logistic regression analysis, variables such as age (*p* = 0.023, odds ratio = 0.87; 95% CI: 0.77–0.98), body weight (*p* = 0.016, odds ratio = 2.12; 95% CI: 1.15–3.89), and CABA status (*p* = 0.0003, odds ratio = 11.9; 95% CI: 3.09–45.82), were correlated with a good clinical outcome in the severe group. These three variables were then subjected to multivariate logistic regression analysis. The results showed that only CABA maintained a statistically positive correlation with good clinical outcomes in cats with moderate or severe azotemia (*p* = 0.003). Cats with CABA were 8.57 times more likely to have a good outcome than those without CABA after hospitalization (Table 8). In the mild group, no statistically significant correlations were detected between the clinical outcome of azotemia and the independent variables (Table 8).

**Table 8.** Univariate and multivariate logistic regression analysis for variables correlated with a good clinical outcome in different groups.

	Severe group				Mild group			
	Univariable analysis		Multivariable analysis		Univariable analysis		Multivariable analysis	
	*p*	OR (95% CI)	*p*	OR (95% CI)	*p*	OR (95% CI)	*p*	OR (95% CI)
Breed (pure to mix)	0.35	1.73 (0.55－5.47)	–	–	0.98	–^a^	–	–
Age	**0.023** *	0.87 (0.77－0.98)	0.17	0.90 (0.78－1.04)	0.34	0.86 (0.64－1.17)	–	–
BW	**0.016** *	2.12 (1.15－3.89)	0.15	1.6 (0.85－3.01)	0.67	0.76 (0.20－2.74)		
Sex (male to female)	0.54	0.69 (0.21－2.25)	–	–	0.54	1.8 (0.09－35.42)	–	–
RS (neutered to intact)	0.28	2.11 (0.54－8.24)	–	–	–^b^	–^b^	–	–
CABA status (absence to presence)	**0.0003** *	11.9 (3.09－45.81)	**0.0034** *	8.57 (2.04－36.05)	0.85	0.75 (0.04－14.58)	–	–
DSA (euhydrated to dehydrated)	0.32	0.53 (0.15－1.88)			0.97	–^c^		
LOS	0.23	1.11 (0.94－1.32)			0.24	1.33 (0.82－2.16)		

^*^ Data showing a statistically significant correlation (*p* < .05, in bold) with clinical outcomes after logistic regression tests.

^a^ Statistical analysis was not available because only one pure cat appeared in the group.

^b^ Statitsical analysis was not available because there was no intact cat in the group.

^c^ Statistical analysis was not available because only two cats were euhydrated in the group. BW, body weight. RS, reproductive status. DSA, dehydration status on admission day. LOS, length of hospital stay. OR, odds ratio. CI, confidence interval.

## Discussion

This study met the hypothesis and illustrated a positive association between renal CABA and good clinical outcomes in patients with moderate or severe azotemia after hospitalization. During hospitalization, azotemic cats in the group with renal CABA presence exhibited a significant decrease in sCr compared to the group without renal CABA presence, and a significantly increased frequency of the presence of renal CABA was demonstrated to be correlated with the classification of decreased sCr percentage. Moreover, cats with azotemia with sCr > 2.8 mg/dl had a high prevalence of good clinical outcomes (21/27, 77.8%) among cats with CABA. However, no associations were found between clinical outcomes and the presence or absence of renal CABA in cats with azotemia with sCr between 1.6 and 2.8 mg/dl. These results suggest that renal CABA could be used as a favorable indicator of good clinical outcomes in cats, particularly for those with azotemia with sCr > 2.8 mg/dl.

The absence of renal CABA has recently been associated with feline CKD and showed a positive correlation with the severity of feline CKD stages (Chou et al. 2021). Generalized increased renal cortical echogenicity was associated with interstitial nephritis, tubular necrosis, and fibrosis confirmed by histopathology (Banzato et al. 2017). These histopathological lesions were considered to be able to eliminate the renal CABA (Chou et al. 2021). Thus, renal CABA may indicate relatively normal cortical structures and potentially lead to improved clinical outcomes after treatment compared to cats with azotemia without renal CABA.

Based on the etiology of azotemia, 22 cats in the severe group were considered to have post-renal azotemia. Most cats (72.7%, 16/22) presented good clinical outcomes after hospitalization. Of the 16 post-renal cats with azotemia with good clinical outcomes, three (18.8%, 3/16) have other underlying intrinsic renal problems. Six (66.7%, 6/9) out of nine post-renal azotemic cats that combined with intrinsic renal azotemia at the same time showed poor clinical outcomes even after hospitalization. Acute post-renal azotemia is rapidly reversible when the origin of urinary obstruction is removed (Langston 2017). This may explain the high percentage of good clinical outcomes in our study for cats with post-renal azotemia without other intrinsic renal diseases (68.1%, 15/22). Fourteen cats with post-renal azotemia with good clinical outcomes after hospitalization had renal CABA (87.5%, 14/16), including three cats with other intrinsic renal problems. The findings in our study indicated that cats with acute post-renal azotemia with renal CABA may have a high probability of decreased sCr levels and good clinical outcomes after hospitalization. This could provide extra information for clinical veterinarians in their decisions to make subsequent treatments.

In the mild group, no association was observed between clinical outcomes and the presence of CABA. Most patients with CABA had sCr levels remaining at CKD stage 2 (8/9, 88.9%) on the day of discharge. Only one subject recovered from azotemia after hospitalization. This result may be because, during the early renal degeneration, the mean gray value of the feline renal cortex did not differ significantly from normal kidneys under ultrasound (Banzato et al. 2017). In early CKD stages (stages 1 and 2), histopathological lesions, such as degeneration, atrophy, inflammation, or fibrosis, accounted for approximately 25%–50% of the renal structure compared with those in stages 3 and 4 with histological lesions greater than 50%–75% (Mcleland et al. 2015). These mild histopathological degenerations may not show increased echogenicity in the renal cortex; however, they can cause mild sCr elevation in cats. This study found a high prevalence of presence of CABA in cats that remained mildly azotemic after hospitalization. Therefore, CABA may not be useful for evaluating clinical outcomes in patients with mild azotemia.

In this study, cats without CABA were older and had lower body weights compared to those with CABA. Furthermore, the sCr levels on the day of discharge were higher in cats without CABA. These trend may be explained by the higher percentage of patients in this study who had a history of CKD. Specifically, of the 29 cats without CABA, 20 (69.0%) had a previous diagnosis of CKD. The association between CABA absence and feline CKD has been reported in previous research (Chou et al. 2021). Additionally, CKD is commonly known as a geriatric disease in cats (Brown et al. 2016), often characterized by increased sCr levels and body weight loss (Freeman et al. 2016). Despite significant differences in age and body weight between cats with and without CABA, our multivariate logistic regression analysis did not reveal a significant correlation between age or body weight and the presence of CABA. Only the presence of CABA was suggested as a factor that correlated with positive clinical outcomes in cats with moderate or severe azotemia, particularly in those with sCr concentrations > 2.8 mg/dl.

Most of the cats in this study were imaged using a linear transducer, with only a few using a micro-convex transducer. The mean gray value may be higher at the regions where the renal tubules are parallel to the incident sound when using a micro-convex transducer. However, no advantages were demonstrated between micro-convex and linear transducers when detecting CABA (Ruth et al. 2013). Using a micro-convex or linear transducer may not have influenced the results of this study.

Uremic cats may exhibit clinical signs such as polyuria, vomiting, and poor appetite. Dehydration caused by either water loss or reduced intake can worsen azotemia. Rehydration was recommended to be conducted over 24-48 h (Sparkes et al. 2016; Polzin 2017). All cats in this study received fluid therapy for over 48 h to ensure they were euhydrated after hospitalization, except for the one that died on the first day of admission. The bias of pre-renal elevation in discharged sCr caused by dehydration appears to be minimalized in this study with the inclusion criteria of at least 48 h of hospitalization.

Only six cats underwent follow-up ultrasound examinations during hospitalization or after discharge. These cats exhibited consistent results in the second examination compared to the initial assessment, suggesting that the CABA status did not change after treatment. However, further investigation is necessary to determine the association between renal disease progression and the presence or absence of CABA.

This study had some limitations that should be considered. Firstly, the sample size was relatively small, which increases the likelihood of type I errors. Secondly, ultrasonography after hospitalization and clinicopathological variables such as phosphate level, urinary protein/creatinine ratio, and symmetric dimethylarginine were not evaluated in this study. The use of sCr as a biomarker for azotemia evaluation was chosen due to its cost-effectiveness, availability, and widespread use in veterinary practice (Kongtasai et al. 2022). While sCr would not be influenced by protein ingestion like BUN, it could be affected by changes in body muscle mass. Cachexia and muscle loss may occur in CKD patients and with long-term confinement in a cage, respectively (Polzin 2017). Unfortunately, data on body condition score (BCS) was not available in this study. Although most cats (63%, 41/65) stayed in the hospital for less than or equal to 7 days (median length of stay), minor effects on sCr due to changes in BCS during hospitalization may still exist. Moreover, putative diagnoses in this study were only based on clinicopathological and imaging findings, and a definitive diagnosis of azotemia may not be possible to make clinically in some cases. In addition, some subjects had only still ultrasonographic images that may have influenced the CABA observation. Finally, renal histopathological examinations were not performed, thus leaving an association between the absence of CABA and unknown histopathological lesions. Despite these limitations, this study offers veterinarians valuable insights into the clinical outcomes of cats with moderate or severe azotemia.

In conclusion, this study found a significant positive correlation between renal CABA and favorable clinical outcomes following hospitalization in cats with moderate or severe azotemia (sCr > 2.8 mg/dl). However, no association was observed between CABA and clinical outcomes in cats with mild azotemia (1.6 < sCr ≤ 2.8 mg/dL). The use of renal CABA in clinical outcome evaluation showed a sensitivity of 80.8% and a specificity of 73.9%. Additionally, intra- and inter-observer agreements in the CABA evaluation demonstrated statistically significant consistencies. These findings suggest that assessing renal CABA may serve as an additional method for evaluating clinical outcomes in cats with moderate or severe azotemia, thus aiding veterinarians in clinical decision-making. However, using CABA assessments in conjunction with clinicopathological examinations, including sCr, urine protein/creatinine ratio, phosphate, and symmetric dimethylarginine, is crucial.

## Data Availability

For any inquiries regarding the data included in the article, please contact the corresponding author.

## Institutional Animal Care and Use Committee (IACUC) or other regulatory body approval declaration

This study was approved by IACUC (No. 109-151 R), National Chung Hsing University.

## Off-label antimicrobial use declaration

Authors declare no off-label use of antimicrobials.

## References

[CIT0001] Banzato T, Bonsembiante F, Aresu L, Zotti A. 2017. Relationship of diagnostic accuracy of renal cortical echogenicity with renal histopathology in dogs and cats, a quantitative study. BMC Vet Res. 13(1):24. doi: 10.1186/s12917-016-0941-z. 28095845 PMC5240265

[CIT0002] Brown CA, Elliott J, Schmiedt CW, Brown SA. 2016. Chronic kidney disease in aged cats: clinical features, morphology, and proposed pathogeneses. Vet Pathol. 53(2):309–326. doi: 10.1177/0300985815622975. 26869151

[CIT0003] Chou PH, Heng HG, Lin FJ, Chen KS. 2021. Absence of renal cortical anisotropic backscattering artifact in feline chronic kidney disease. Vet Q. 41(1):210–216. doi: 10.1080/01652176.2021.1941397. 34112054 PMC8245094

[CIT0004] Debruyn K, Haers H, Combes A, Paepe D, Peremans K, Vanderperren K, Saunders JH. 2012. Ultrasonography of the feline kidney: technique, anatomy and changes associated with disease. J Feline Med Surg. 14(11):794–803. doi: 10.1177/1098612X12464461. 23087005 PMC11112170

[CIT0005] Dibartola SP, Westropp JL. 2020. Chapter 38: clinical manifestations of urinary disorders. In: Nelson RW, Couto CG, Couto KM, eds. Small animal internal medicine. 6th ed. St. Louis, MO: Elsevier. p. 649–739.

[CIT0006] Freeman LM, Lachaud MP, Matthews S, Rhodes L, Zollers B. 2016. Evaluation of weight loss over time in cats with chronic kidney disease. J Vet Intern Med. 30(5):1661–1666. doi: 10.1111/jvim.14561. 27527534 PMC5032880

[CIT0007] Kongtasai T, Paepe D, Meyer E, Mortier F, Marynissen S, Stammeleer L, Defauw P, Daminet S. 2022. Renal biomarkers in cats: a review of the current status in chronic kidney disease. J Vet Intern Med. 36(2):379–396. doi: 10.1111/jvim.16377. 35218249 PMC8965260

[CIT0008] Lamb CR, Dirrig H, Cortellini S. 2018. Comparison of ultrasonographic findings in cats with and without azotaemia. J Feline Med Surg. 20(10):948–954. doi: 10.1177/1098612X17736657. 29019448 PMC11129239

[CIT0009] Landis JR, Koch GG. 1977. The measurement of observer agreement for categorical data. Biometrics. 33(1):159–174. doi: 10.2307/2529310. 843571

[CIT0010] Langston CE. 2017. Chapter 322: acute kidney injury. In: Ettinger SJ, Feldman EC, Côté E, eds. Textbook of veterinary internal medicine. 8th ed. St. Louis, MO: Elsevier. p. 4650–4685.

[CIT0011] Mcleland SM, Cianciolo RE, Duncan CG, Quimby JM. 2015. A comparison of biochemical and histopathologic staging in cats with chronic kidney disease. Vet Pathol. 52(3):524–534. doi: 10.1177/0300985814561095. 25516066

[CIT0012] Polzin DJ. 2017. Chapter 324: chronic kidney disease. In: Ettinger SJ, Feldman EC, Cote E, eds. Textbook of veterinary internal medicine. 8th ed. St. Louis, MO: Elsevier. p. 3305–3330.

[CIT0013] Ruth JD, Heng HG, Miller MA, Constable PD. 2013. Effect of anisotropy and spatial compound imaging on renal cortical echogenicity in dogs. Vet Radiol Ultrasound. 54(6):659–665. doi: 10.1111/vru.12056. 23763283

[CIT0014] Siddappa JK, Singla S, Al Ameen M, Rakshith SC, Kumar N. 2013. Correlation of ultrasonographic parameters with serum creatinine in chronic kidney disease. J Clin Imaging Sci. 3:28. doi: 10.4103/2156-7514.114809. PMC3779384 24083065

[CIT0015] Sparkes AH, Caney S, Chalhoub S, Elliott J, Finch N, Gajanayake I, Langston C, Lefebvre HP, White J, Quimby J. 2016. ISFM consensus guidelines on the diagnosis and management of feline chronic kidney disease. J Feline Med Surg. 18(3):219–239. doi: 10.1177/1098612X16631234. 26936494 PMC11148907

[CIT0016] Yabuki A, Endo Y, Sakamoto H, Nagayoshi T, Matsumoto M, Suzuki S. 2008. Quantitative assessment of renal cortical echogenicity in clinically normal cats. Anat Histol Embryol. 37(5):383–386. doi: 10.1111/j.1439-0264.2008.00866.x. 18513275

[CIT0017] Zotti A, Banzato T, Gelain ME, Centelleghe C, Vaccaro C, Aresu L. 2015. Correlation of renal histopathology with renal echogenicity in dogs and cats: an ex-vivo quantitative study. BMC Vet Res. 11(1):99. doi: 10.1186/s12917-015-0415-8. 25909709 PMC4413530

